# Semitendinosus vs quadriceps tendon autograft in anterior cruciate ligament reconstruction (SEQUAR): protocol for a prospective randomized controlled trial

**DOI:** 10.1186/s12891-025-09183-w

**Published:** 2025-10-13

**Authors:** Daniel Castellanos Dolk, Vasileios Sarakatsianos, Tobias Wörner, Mikael Östin, Riccardo Cristiani, Joanna Kvist, Anders Stålman

**Affiliations:** 1 Department of Molecular Medicine and Surgery, Stockholm Sports Trauma Research Center, Karolinska Institutet, Stockholm, Sweden; 2 Department of Orthopaedic Surgery, Capio Specialistvård Motala, Motala, Sweden; 3 Capio Artro Clinic, FIFA Medical Centre of Excellence, Sophiahemmet Hospital, Stockholm, Sweden; 4 Artro Clinic, FIFA Medical Centre of Excellence, Sophiahemmet Hospital, Stockholm, Sweden; 5 Unit of Physiotherapy, Department of Health, Medicine and Caring Sciences, Linköping University, Linköping, Sweden

**Keywords:** Orthopaedics, Anterior cruciate ligament (ACL) reconstruction, Knee, Quadriceps tendon graft, Semitendinosus tendon graft

## Abstract

**Background:**

Anterior cruciate ligament (ACL) rupture is a devastating injury for most individuals, especially in athletes with the desire to go back to pivoting sports. In ACL reconstruction (ACL-R), multiple autograft options are available, each with distinct advantages and limitations. The quadriceps tendon (QT) autograft has gained increasing attention due to its favorable biomechanical properties and lower donor site morbidity, yet studies comparing only athletic populations remains limited. This study protocol describes a randomized controlled trial (RCT) aiming to compare QT autograft with bone-block (QT-B) with semitendinosus tendon (ST) autograft in athletes undergoing primary ACL-R.

**Methods:**

This is a single center RCT. Two Hundred ACL-R patients aged 16–40 years with a Tegner Activity Scale (TAS) of ≥ 7 prior to the ACL injury will be randomized to receive either a ST graft or a QT-B graft at the time of ACL-R. All participants will receive standard postoperative care and rehabilitation, followed by comprehensive follow-up. The primary outcome was the side-to-side (STS) difference in anterior tibial translation (ATT) at 6 months. Secondary outcomes include patient-reported outcome measures (PROMs), isokinetic strength testing, imaging-based graft evaluation, surgical and clinical events, and 10-year radiographic evidence of joint degeneration. Detailed definitions and timepoints are provided in the full manuscript (Table 1).

**Discussion:**

This protocol is the first RCT comparing the ST autograft to the QT-B autograft exclusively in athletes with TAS ≥ 7 undergoing ACL-R. In addition, this trial has the ambition of including 200 patients, thereby making it the largest trial to compare these two grafts to date. The successful completion of the trial has the potential to change current practice and contribute to the existing knowledge regarding graft choice in athletes undergoing ACL-R.

**Trial registration:**

The trial is registered at ClinicalTrials.gov: NCT04295148.

Registered date: 4 Mar 2020 (retrospectively registered).

## Background

Anterior cruciate ligament (ACL) ruptures are one of the most feared and potentially career-ending injuries for athletes. A recent Swedish study reported that female athletes may experience up to a 24-fold higher incidence of ACL reconstruction (ACL-R) compared to females in the general population, and male athletes up to a ninefold increase [1]. For those aiming to return to high-demand pivoting sports or activities, ACL-R is typically recommended as the preferred treatment to enable a safe and effective return to sports. The primary objective of surgical treatment with ACL-R is to restore anterior knee laxity [2], which can objectively be assessed by measuring anterior tibial translation (ATT) with an arthrometer such as KT-1000 [3]. Assessment of ATT using the KT-1000 arthrometer is best performed through the side-to-side (STS) difference, which is the standard method for detecting clinically relevant knee laxity and has demonstrated reliable and reproducible measurements for evaluating outcomes after ACL reconstruction [4].

The most common choice of graft for ACL-R in Sweden and worldwide is the hamstring tendon (HT)autografts, more particularly the semitendinosus tendon (ST) autograft [5, 6]. Bone-patellar tendon-bone (BPTB) autografts are another well-known and frequently used option. BPTB autografts have been associated with lower rates of graft failure and anterior knee laxity, but carry a higher risk of anterior knee pain, kneeling discomfort, and donor-site morbidity [7, 8]. In contrast, HT autografts typically result in less anterior knee pain and fewer harvest-site complications, although they have been associated with greater postoperative ATT and higher graft failure rates compared to BPTB [9, 10]. Despite these biomechanical concerns for HT autografts, recent evidence suggests that isolated semitendinosus (ST) grafts are less prone to increased laxity and are not significantly affected by factors such as graft diameter [11, 12].

Interestingly, the quadriceps tendon (QT) autografts yields comparable clinical and functional outcomes to both HT and BPTB autografts but with certain unique advantages [13, 14]. Notably, QT autografts have been associated with reduced incidence of anterior knee pain and kneeling discomfort compared to BPTB grafts, two common complaints after ACL-R with BPTB grafts [15, 16]. Moreover, QT autografts might offer the lowest donor-site morbidity among the three autografts (HT, BPTB and QT grafts), making it an interesting option to use [17].

The quadriceps tendon (QT) can be harvested either as an all–soft tissue graft (QT-S) or with a patellar bone block (QT-B). QT-B offers the theoretical advantage of bone-to-bone healing, potentially improving graft incorporation and stability, although there is a risk of complications such as patellar fracture. In contrast, QT-S avoids bone harvest and may be more suitable in certain patient groups, such as skeletally immature individuals [18, 19]. Recent systematic reviews and registry studies have reported comparable clinical outcomes between QT-B and QT-S [18, 19], although QT-B remains the more frequently studied QT autograft [20].

High quality registry studies have also provided conflicting evidence on whether QT grafts are associated with higher revision rates. In the Danish Knee Ligament Reconstruction Registry there have been reports that the revision rate for QT autografts was higher than that for HT and BPTB grafts; however, these findings have later been shown to only implicate cases where surgeons with less experience using the QT autografts have performed the ACL-R, emphasizing the importance of surgical expertise and proper technique in achieving optimal outcomes [21, 22].

Several randomized controlled trials have compared QT-B and HT grafts, showing no significant differences in knee laxity or subjective outcomes at 12 and 24 months postoperatively [13, 23, 24]. However, other functional outcomes will differ and are also clinically relevant when comparing QT and HT autografts. Both rehabilitation and muscle strength recovery after ACL-R will be affected by the graft type chosen for the procedure. For example, individuals undergoing ACL-R with QT graft have greater knee flexion strength but lesser knee extension strength compared to HT grafts [24, 25]. Likewise, isokinetic quadriceps strength measurements at 7 months have shown that patients undergoing ACL-R with QT grafts have impaired isokinetic quadriceps strength compared to HT and BPTB grafts [26]. Quadriceps strength after ACL-R with QT grafts often remains suboptimal for up to 24 months, which could be a reason for delay of return to sport. However, hamstring strength recovery is consistently better preserved with QT grafts than with HT grafts. Therefore, the rehabilitation protocol should be customized to address these deficits, prioritizing adequate quadriceps recovery to reduce the risk of reinjury while taking advantage of the hamstring strength preservation provided by QT grafts [27].

While the existing literature provides valuable insights into the outcomes of QT and HT grafts, a large RCT comparing ST and QT-B autografts in athletes with extended follow-up in a large range of outcomes measures, such as objective knee laxity and patient reported outcome measures (PROM) is essential to add to the current knowledge about the optimal graft choice for athletes.

This protocol has been prepared following the Standard Protocol Items: Recommendations for Interventional Trials (SPIRIT) 2013 Statement to ensure transparency, comprehensiveness, and adherence to best practices in protocol development [28].

## Methods

### Objectives

The primary aim of this study is to investigate potential differences in STS knee laxity at 6 months after ACL-R performed with either a ST graft or a QT-B graft in athletes with Tegner Activity Scale (TAS) ≥ 7 [29]. Patients will be followed for 6 months post ACL-R for the primary endpoint. Various secondary outcomes will also be measured to enable comprehensive evaluation of both short- and long-term outcomes.

### Hypothesis

For primary outcomes, it is hypothesized that 1) there will not be any significant differences in STS knee laxity at 6 months.

Further, for secondary outcome, it is hypothesized that 2) deficits in the isokinetic knee extension and flexion strength will differ between the choice of graft, 3) no differences in hop test LSIs will be observed, 4) no differences in frequently used PROMs after ACL-R will be noted, 5) no differences in return to sports rates, 6) nor differences in graft site morbidity will be seen.

Finally, it is hypothesized that 7) no differences in graft failure or contralateral ACL rupture will be observed, and that 8) the incidence of radiographic or symptomatic (PROMs) osteoarthritis at 10 years will be similar across both grafts.

### Study design

This is a single-center prospective randomized controlled trial.

### Participants

The study will take place at the Capio Artro Clinic, Stockholm, Sweden, a highly specialized orthopedic clinic performing over 1000 ACL-Rs annually. The recruitment of the study participants will be performed at the outpatient department once inclusion and exclusion criteria have been checked. The TAS level will be assessed by the orthopedic surgeon responsible for patient enrolment into the study. The patients meeting inclusion criteria will be informed about the study and will be asked to participate. During the outpatient visit they will be handed the written consent form by the orthopedic surgeon and will later be randomized by an independent researcher, not directly involved in the study. All participants will undergo a standardized preoperative rehabilitation program prior to ACL-R. Data collection for the primary outcome will continue for two years.

Inclusion criteria:

- Age 16 −40 years
- Pre-injury TAS ≥ 7
- Intended return to sports to prior sport and TAS Level
- Time between injury and inclusion not more than 6 months
- MR verified ACL rupture

Exclusion criteria:

- Previous knee injury with symptoms before ACL injury
- Neurological disease, inflammatory disease, connective tissue disease or balance disorder
- Previous lower limb fracture or surgery
- Laxity in the medial collateral ligament (MCL) and lateral collateral ligament (LCL) < grad 1
- Posterior cruciate ligament (PCL) rupture or Multiligament knee injury (MLKI)
- Radiographic sign of osteoarthritis (OA)
- Previous knee surgery or ligament injury in contralateral knee
- Beighton score ≥ 5

### Intervention

Patients will be randomized to ACL-R with either ST autograft (n = 100) or QT-B autograft (n = 100).

### Sample size calculation

To ensure adequate power, the sample size is based on Capio Artro Clinic’s clinical registry database from KT-1000 anterior knee laxity measurements available at the time of study planning, which indicated a standard deviation (SD) of 2.3 mm. This database includes over 7000 ACL-R and has served as the source to study postoperative knee laxity, functional test and graft comparisons [10–12, 30–32]. The power analysis is structured as follows: a 5% significance level, 80% power, and a minimum detectable difference of 1 mm in KT-1000 anterior knee laxity measurements. Based on these parameters, a minimum of 85 participants per group will be required as adequate sample. To account for potential dropouts, the sample size will be increased by 15%, resulting in 100 participants per group or 200 in total [33]. Recruitment will continue until each group has 100 fully evaluable participants for primary outcome measures.

### Randomization

In this single-center study, we will employ a simple 1:1 randomization to allocate participants to the two groups. The randomization process will be conducted by the independent researcher (not involved in the study) using randomizer.org, an online platform that generates random assignments. Participants will be randomized roughly one week after the patients sign the study consent form to participate in the study. Once included and randomized, the participants will fill out the baseline preoperative PROMs questionnaires. Given the inherently different graft harvesting sites, blinding of study participants, orthopedic surgeons as well as physiotherapist is not considered feasible. Participants will be informed of their allocation on the day of surgery, unless they explicitly requested to receive this information earlier. This approach was chosen to minimize potential cross-overs or withdrawals.

**Fig. 1:**
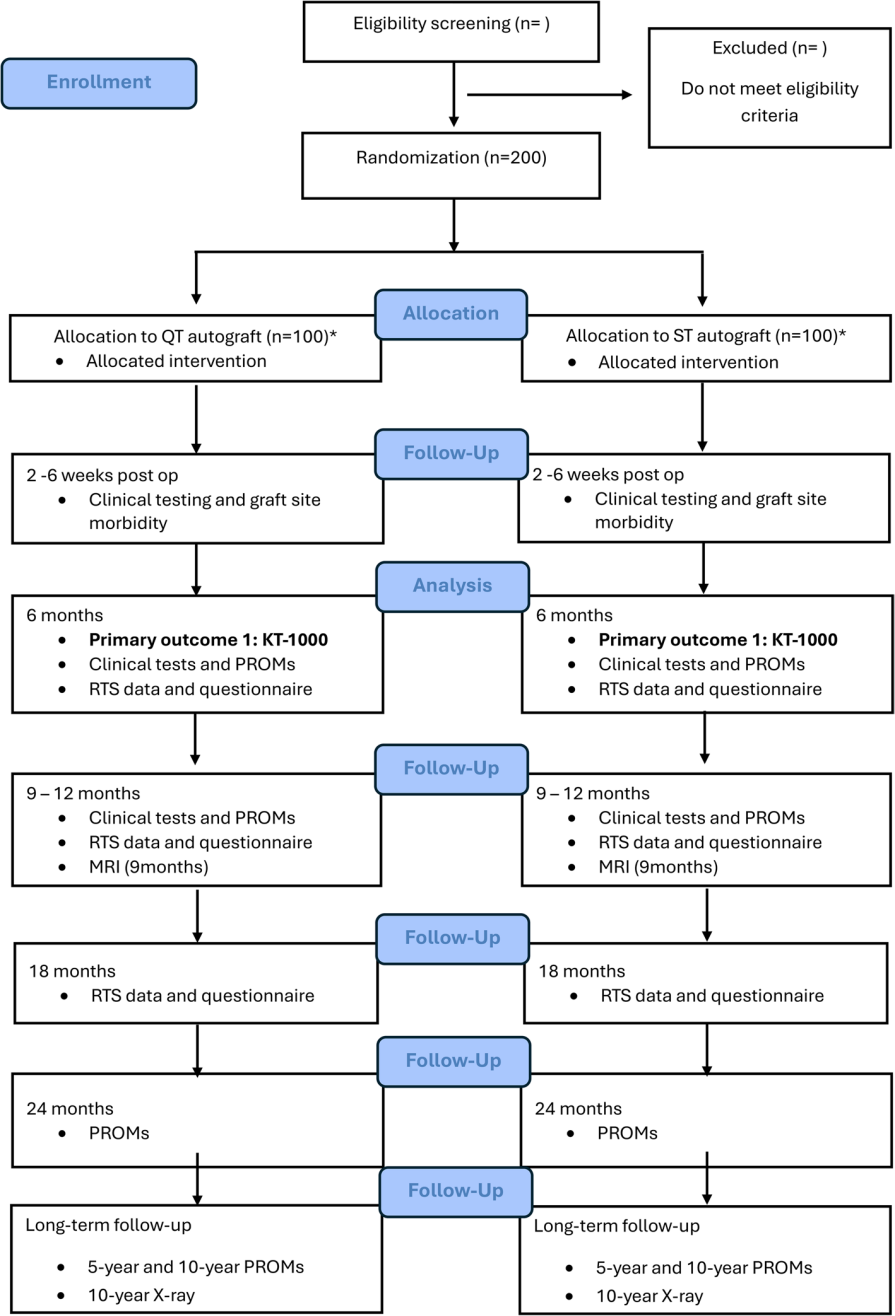
Planned CONSORT flow diagram for the randomised trial protocol

Although no stratification for sex will be performed, our aim is to have a balanced representation of sex that reflects the typical patient population for this condition at the clinic, with a distribution of 40% females and 60% males across both groups. This ratio also considers known differences in re-rupture risk and subjective outcome scores between sexes. Simple randomization in a RCT with 200 or more participants is well-suited as the likelihood of significant treatment imbalance is often negligible at these sample sizes [34]. All randomization procedures and study design elements are structured in accordance with CONSORT guidelines to ensure the highest standards of methodological transparency and rigor. Figure 1 shows the planned CONSORT flowchart.

### Surgical procedures

All surgeries will be performed by one of four experienced surgeons (annual caseload > 50 ACL-R/revisions and total case history of > 500 ACL-R/revisions) [35], with vast experience using both ST and QT-B autografts. All surgeons were trained within the same clinic and follow a standardized institutional ACL reconstruction protocol. All patients will follow Capio Artro Clinic’s standardized anesthesia protocol and will be operated in a standard supine position. Patients will receive a preoperative antibiotic prophylaxis (2 g of Cloxacillin I.V.) which will be administered 30–45 min prior to surgery.

A standard diagnostic arthroscopy will be performed to map all the injuries. Meniscal pathology, including radial tears, ramp lesions, and root injuries, will be treated as clinically indicated with repair or resection depending on the type of injury and whether there are conditions for healing, and these procedures will not be considered exclusion criteria. Cartilage damage will be documented according to the International Cartilage Repair Society (ICRS) Classification [36] and handled according to the usual routine depending on size and depth. Superficial or minor cartilage injuries will be left while larger and deeper damaged cartilage will typically be drilled with a 1.6mm drill bit to stimulate cartilage regeneration that can cover the defect (microdrilling).

The harvest of the quadriceps tendon will be performed from a 5 cm long incision made from the middle of the patella to proximal. A full-thickness quadriceps tendon graft will be harvested from the middle section of the quadriceps tendon with a width of between 8–10 mm and with approximately 1 × 2 cm bone block from the proximal patella. The Tight Rope BTB I®(Arthrex®) will be used on the bone-block, and at the other tendinous end, two different non-reabsorbable sutures (either Ethibond® or Fiberwire®, size 2) will be sutured into the graft using baseball stitches.

The harvest of the semitendinosus tendon will be performed by using a 3–4 cm oblique incision over the pes anserinus. Using a Semitendinosus Tendon Stripper® (Arthrex®), the semitendinosus tendon will be harvested and will be four-folded. In case the graft volume is too small (< 8 mm), an additional gracilis tendon graft will be harvested and added according to the usual routine. The four-folded semitendinosus tendon graft will be looped over the Tight Rope I ® (Arthrex®) with Ethibond® size 5 and one non-reabsorbable sutures (either Ethibond® or Fiberwire®, size 2) into each of the two distal ends (free tails) of the ST graft using baseball stitching.

The selected autograft will be soaked in a sterile vancomycin solution (5mg/mL) prior to implantation during ACL-R to provide localized antibiotic prophylaxis. This technique aims to reduce the risk of postoperative infections by delivering high local concentrations of vancomycin directly to the graft and the knee joint [37–39].

In both groups, anatomical reconstruction of the ACL is performed with the accessory anteromedial portal in a in-side out technique through this portal the surgeon will use a 1.6 mm drill bit guide that will be placed and drilled in an anatomic position behind the lateral intercondylar ridge, approximately 60% of the anterior posterior width of the femur, toward the anterior medial (AM) bundle footprint, and later over-drilled to the same diameter as that of the graft. In the tibia, the tibia guide will be set at 50–55 degrees, depending on the patient anatomy, and once again a 1.6 drill bit guide will be drilled in the middle of the tibial ACL footprint, and over-drilled to the same diameter as that of the graft.

Both grafts used in this study will be fixed with Milagro® interference screw in the tibia with at least 1 mm oversized compared to the graft. Fixation distally in the tibia occurs in the degree of flexion that the surgeon decides optimal for graft isometry throughout the whole range of motion (ROM) of the knee.

Lateral extra-articular tenodesis (LET) procedures will not be performed in this cohort. Individuals with generalized joint hypermobility (Beighton score ≥ 5), who may have been considered for LET, will be excluded.

### Postoperative rehabilitation

For both surgical procedures (ACL-R with ST or QT-B grafts), patients will be followed by a physiotherapist that will follow the standardized rehabilitations protocols from the clinic and individualize the rehabilitation when needed for the specific patient. Participants will be allowed to fully weight bear the knee with the aid of crutches postoperatively in the event of isolated ACL-R and in ACL-R with meniscal resection, meniscal ramp lesion repair or meniscal bucket-handle repair. Individuals undergoing meniscal root repair will be allowed to partially weight bear for 6 weeks and those undergoing a repaired radial meniscal lesion will be instructed not to weight bear during the first 6 weeks. ROM will be restricted for all meniscal repair procedures initially to protect the meniscus early in the healing process. Surgically treated cartilage defects in the femuro-patellar region will be allowed to weight bear in the ROM restricted at 0–30 degrees with a knee orthosis, while surgically treated cartilage defects in the tibiofemoral region will be treated with partial weight bearing for 6 weeks. Postoperative weight-bearing and ROM restrictions will not be used as stratification variables, and no subgroup analyses will be performed based on these factors.

According to the standardized rehabilitation protocol, the rehabilitation progression follows a structured, criterion-based protocol rather than a purely time-based schedule. After the initial six weeks, patients transition through defined phases focusing on strength development, neuromuscular control, and functional capacity. From week 13–14 onward, there will be no restrictions on quadriceps training. Clearance to begin impact activities such as running will typically occur around 4 to 5 months postoperatively and requires ≥ 80% limb symmetry index (LSI) in quadriceps and hamstring strength, along with resolution of joint effusion. Return to sport (RTS) is generally assessed between 8 and 12 months and requires ≥ 90% LSI in both strength and hop testing, absence of swelling or functional complaints, and adequate movement quality during sport-specific tasks. Although adherence to the rehabilitation program will not be tracked, all functional tests are performed at standardized checkpoints using validated protocols to assess progression (Table 1). Tailoring of the rehabilitation program will be allowed based on the individual recovery and sport-specific demands.

**Table 1:**
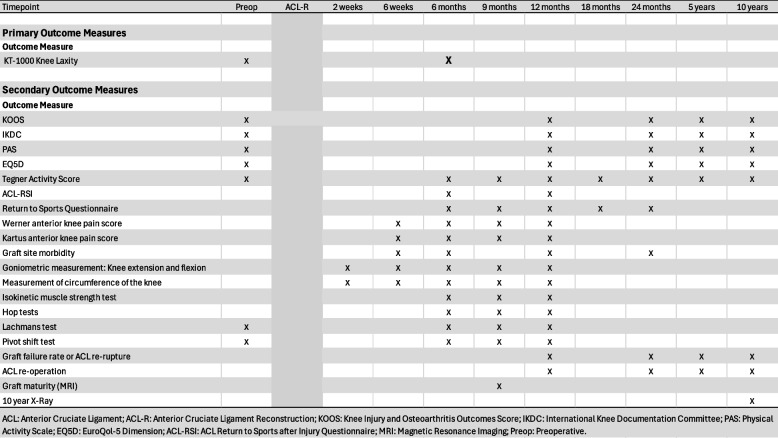
Scheduled follow-up and assessment of primary and secondary outcomes

### Outcomes

The rationale behind the outcome measurement in this study is to evaluate the patients knee laxity at 6 months, subjective knee function through PROMs, as well as other physical and objective outcomes. The exact time points of the different outcome measurements during the outpatient follow up are summarized in Table 1.

### Primary outcome

#### Knee Laxity at 6 months post ACL-R

Knee Laxity will be measured preoperatively and at 6 months postoperatively using the KT-1000 arthrometer® (MEDmetric®, Corp, San Diego, CA, USA) in accordance with the clinic’s standard routine for postoperative evaluation of ACL-R [10]. Patients will be positioned supine with the knee at approximately 20 degrees of flexion with the help of a bolster placed under the thighs, and with the feet are supported on a footrest to minimize muscle contraction and prevent external rotation. The contralateral (uninjured) knee will be tested first, followed by the ACL-injured knee. For each knee, three trials will be conducted using the standardized anterior force corresponding to 134 N (third audible “click” of the KT-1000). Between trials, the arthrometer will be recalibrated and the median value of the three valid measurements will be recorded for each side. The STS difference in ATT between the ACL-injured knee and the healthy contralateral knee (measured preoperative to postoperative) will be expressed in millimeters. To reduce inter-rater variability, all measurements will be performed by two unblinded, experienced physiotherapists, each with over 10 years of experience in arthrometric laxity assessment following ACL injury and reconstruction.

### Secondary outcomes

#### Subjective knee function

The PROMs and the specific time points of measurement are presented in Table 1.

- The Knee injury and Osteoarthritis Outcome Score (KOOS) [40],
- The International Knee Documentation Committee Subjective Knee Form (IKDC-SKF) [41],
- The Physical Activity Scale (PAS) [42],
- The the EuroQol 5-Dimension score (EQ-5D) [43],
- The Tegner Activity Scale [29],
- The Anterior Cruciate Ligament Return to Sports after Injury (ACL-RSI) [44],
- The Werner anterior knee pain (AKP) score [45],
- The Kartus anterior knee pain score [46]
- Graft site morbidity questions
- Return to Sports questionnaire (Table 2)

All scores that will be used have either originally been created or translated to Swedish [47, 48].

### Objective knee function

#### Knee joint examination

All clinical examination will be performed by one of the four experienced orthopedic knee surgeons participating in the study. The knee examination will assess knee ROM using a goniometer [49] and knee joint effusion (circumference of the joint using a measuring tape centered at the center of the patella) [50], anterior knee laxity with the knee in 20–30 ^o^ flexion (Lachman’s test), rotational laxity using the pivot shift test.

#### Graft site morbidity

Graft site morbidity will be assessed using two dichotomous (yes/no) questions. The first question will determine whether participants experience reduced sensation around the surgical site. If they answer 'yes,' the affected area will be measured in cm^2^. The second question will assess whether participants experience pain or discomfort at the surgical site.

#### Functional knee testing

To assess functional outcomes after ACL-R, isokinetic muscle strength testing and two single-legged hop (one-leg hop for distance and cross over hop) tests will be used [51]. Following a 10 min self-paced warm up in a stationary cycle ergometer, patients will perform the isokinetic strength tests and subsequently the hop tests.

The limb symmetry indexes (LSIs) of the peak extension and flexion torque and single-leg-hop tests are calculated as [involved limb/uninvolved limb × 100] for each test. For isokinetic strength and single-leg hop tests, symmetrical performance is defined as achieving at least 90% of the contralateral leg’s performance (LSI ≥ 90%) [31, 32]. Patients who achieve an LSI ≥ 90% in all four tests (isokinetic quadriceps strength, isokinetic hamstring strength, and single-leg hop test) are considered to have restored symmetrical knee function [31].

#### Isokinetic muscle strength testing

Isokinetic testing will be performed using the Biodex System 3 dynamometer (Biodex Medical Systems, Shirley, New York, USA) at follow-up time outlined in Table 1 [52]. The test will be conducted within a ROM from 90° to 0° of knee flexion, starting with the uninjured knee. The testing procedure will be verbally explained to participants, and they will be allowed two to three practice trials to familiarize themselves with each part of the protocol. Concentric knee extension and flexion torque will be measured bilaterally at 90°/s (5 test repetitions) and 240°/s (10 test repetitions), and isokinetic eccentric knee extension and flexion torque will be measured at 90°/s (5 test repetitions), according to the clinic’s previously published standard routine [26]. Standardized, verbal encouragement is provided throughout the test, and the peak extension and flexion torque values (highest recorded torque) are documented.

#### Hop testing

Hop performance will be assessed by the Single-Leg Hop test for Distance and the Crossover Hop test for Distance [51]. The Single-Leg Hop test for Distance is performed by standing on one leg and jumping straight ahead as long as possible, with hands free for balance, and landing on the same leg. The Crossover Hop test for distance entails three consecutive forward hops while alternately crossing over a 15 cm marked line on the floor (crossing the line medially) before landing on the same leg. After verbal instruction and practice, participants will perform three valid hop trials per leg, starting with the uninjured side. Trials are considered valid with a stable landing; attempts with balance loss or compensatory steps are repeated. The best distance (toe-to-heel) is recorded for analysis. Participants who have an isokinetic concentric knee extension torque LSI of < 70%, feel insecure about it, or have not reached the rehabilitation stage in which plyometrics are practiced will be excluded from hop-testing.

#### Time to return to sports rates

A Return to Sports Questionnaire (Table 2), adapted from the Oslo Sports Trauma Research Center (OSTRC) Overuse Injury Questionnaire [53], will be used to evaluate participants' return to sport or physical activity at various time points during the follow-up period, as outlined in Table 1.

Participants will first be asked whether they have returned to the same sport or physical activity they engaged in prior to their injury. Those who indicate that they have returned, will further be asked to specify their level of participation, according to the first question of the OSTRC questionnaire (Table 2). For participants who have not returned to their previous activity, an additional question not belonging to the OSTRC exploring the primary reasons for non-returning to sports will be asked. Options that will be included are undergoing rehabilitation, experiencing poor knee function, lacking confidence or trust in the knee, fear of reinjury, or other reasons, with space provided for elaboration.

**Table 2:**
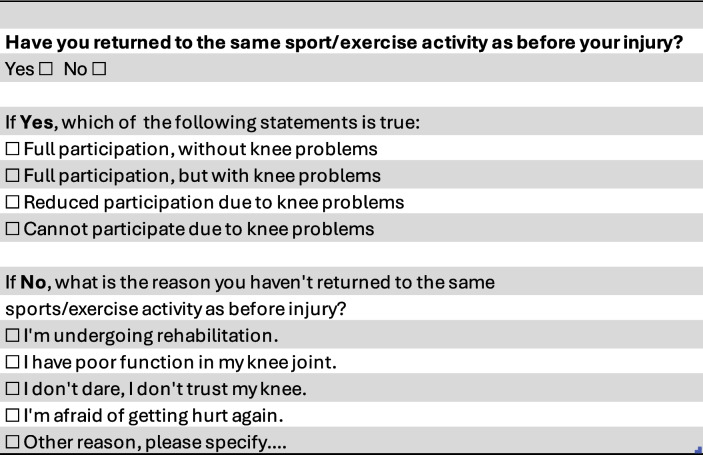
Return to sport questionnaire

The data collected will be used to assess return-to-sport rates, levels of participation, and factors influencing the decision not to return. This information is critical for identifying areas where additional support or targeted interventions may help optimize outcomes for individuals recovering from knee injuries.

#### Graft failure, revision ACL-R and other adverse events

Participants will be followed longitudinally for a period of 10 years to monitor ACL graft failure (recurrent instability requiring revision), ACL re-rupture rates, and other adverse event affecting outcome (contralateral ACL rupture, infection or septic arthritis, bleeding, and any reoperation performed during the study period). Clinical and surgical records will be reviewed, and participants will be contacted periodically according to Table 1 to assess their knee health and report any additional surgeries or injuries. This long-term follow-up aims to evaluate the durability of ACL-R and identify factors associated with graft failure and re-rupture, such as patient demographics, activity levels, or surgical techniques. At the 5- and 10-year follow-ups, any undocumented events will be retrospectively collected via the Swedish National Knee Ligament Register (SNKLR).

#### Imaging

Participants will undergo imagining assessment after ACL-R. Participants will undergo MRI (Magnetic resonance imaging) scans at 9 months postoperatively to assess graft Maturity. MRI will be performed with 1.5-T with standard oblique coronal proton density-weighted projection with 3 mm slices as previously described by Ma et al. [54]. Signal-to-noise quotient (SNQ) values will be measured in coronal oblique projections to visualize the entire graph. The maturity of the graft will be measured in three parts of interest: (1) the proximal part, (2) the middle part, and (3) the distal part, as described by Ma et al. [54]. Also, customary coronal, sagittal and axial projections will be done to evaluate other anatomical and operative factors. MRI will be performed at 9 months postoperatively to assess the graft maturity at the time to usual RTS evaluation.

To assess the development of OA 10 years after ACL-R, all participants will undergo bilateral knee weight-bearing radiographs. Radiographs will be obtained in standardized weight-bearing projections, including anterior–posterior and lateral views, as well as a skyline Merchant view of the patellofemoral joint, to comprehensively evaluate joint space narrowing, osteophyte formation, subchondral sclerosis, and other radiographic features of OA. Grading will be performed using the Kellgren-Lawrence classification system, a widely recognized method for assessing the severity of OA [55].

#### Statistical analysis

The statistical analysis will be conducted using SPSS software (version 29.0 or updated version). Descriptive statistics, including frequency, mean, and standard deviation, will be computed for all variables, and normal distributions will be assessed using histograms, Q-Q plots and Kolmogrov-Smirinov test. Continuous variables will be compared using parametric or non-parametric methods, depending on data distribution. Categorical variables will be analysed using Pearson’s chi-square test or Fisher’s exact test.

For anterior knee laxity measurements (KT-1000), mean ± standard deviation and median (range) values will be reported. A variance-based statistical test will be used to compare, preoperative and postoperative anterior knee laxity, as well as ATT reduction between treatment groups. The choice of test will be determined based on the distribution and characteristics of the data after collection. Adjustments for baseline values may be included as part of a secondary analysis to ensure robust comparisons.

Postoperative STS values in knee laxity will be categorized and presented in three groups according to the IKDC form: Normal ≤ 2 mm (A), nearly normal 3–5 mm (B), and abnormal > 5 mm (C and D) [10, 11, 56]. The postoperative side-to-side (STS) difference will be dichotomized as either normal (≤ 5 mm) or failure (> 5 mm). Distributions and proportions of failures will be compared between intervention groups using Pearson’s chi-square test.

The study will employ both intention-to-treat (ITT), and per-protocol (PP) to ensure robust assessment of outcomes for each surgical group. The ITT analysis will include all randomized patients in their originally assigned groups, regardless of any protocol deviations or dropouts. Missing data for the ITT analysis will be managed using imputation methods where necessary. The PP analysis will include only those patients who fully adhered to the study protocol, completing their assigned surgery and primary outcome assessments.

Secondary outcomes will be evaluated across follow-up timepoints (Table 1). Continuous outcomes will be analyzed using a variance-based statistical test to assess within-group changes over time. Adjustments for baseline values may be included as part of a secondary analysis to ensure robust comparisons. Individual time points will be assessed using independent samples t-tests or non-parametric test, depending on the distribution of the data.

Categorical outcomes will be analyzed using Pearson’s chi-square or Fisher’s exact tests. Logistic regression may also be used to adjust for potential confounders when analyzing binary outcomes. Time-to-event outcomes, such as graft failure and return to sport, will be analyzed using Kaplan–Meier survival curves and Cox proportional hazards regression models, assuming the proportional hazards assumption is met.

## Discussion

The decision regarding the type of graft to use for ACL-R in athletes is complex and requires careful consideration, and should be based on the athlete’s specific sporting demands as well as the surgeon’s experience and preferences [57]. Despite growing interest in QT autografts, clinical evidence comparing QT to HT autograft, especially in athletic populations, remains limited. Few RCTs have studied graft choice for athletes undergoing ACL-R, especially when exploring knee laxity and long-term outcomes. Given the increasing use of QT autografts in ACL-R, a study comparing it with most common autograft in athletes is timely and relevant.

This study protocol describes a prospective RCT following ACL-R with either ST or QT-B autografts in athletes with TAS ≥ 7. This is the first trial to exclusively include athletes with TAS ≥ 7 and with the documented desire to go back to their preinjury TAS level after ACL-R. Also, with the ambition to include 200 patients, this will be the largest study to compare the ST autograft to the QT-B autograft in ACL-R in a RCT [58].

The primary outcome described in this study protocol is anterior knee laxity objectively measured by the KT-1000 at 6 months. This study will provide important clinical data regarding possible differences between both autografts. Although QT-B and ST autografts have structural differences, the current evidence has failed to show differences in postoperative anterior knee laxity between QT and HT autografts. The inclusion of a various secondary outcomes, including other PROMs, other functional test, return-to-sport timing, graft failure or contralateral ACL injury, and radiographic osteoarthritis, will provide a wider view of the effects of graft choice in this group of TAS ≥ 7 athletes.

QT-B and ST autografts should differ in their biomechanical and functional implications, particularly with regard to postoperative muscle strength. Likewise, no consistent differences have been reported in PROMs between the two grafts [58, 59]. QT autografts are associated with a greater impact on quadriceps strength, while ST autografts more commonly affect postoperative hamstrings function [25, 26]. Current evidence supports the presence of significant differences between the two autografts, however how these differences influence functional outcomes in athletes with the intended aim of returning to their pre-injury level of sport is still poorly understood.

Following various outcome after ACL-R over time in both QT-B and ST autograft groups is also crucial for assessing short-term and long-term consequences of graft selection. Functional tests and RTS may provide insight into the early recovery and performance, while graft failure or contralateral injury reflects the durability and risk of new injuries. Although this trial is not designed to evaluate long-term outcomes, it may provide some insights into the long-term effects of different autografts in athletes aiming to return to sport.

Study limitations include the unblinded design for patients that were deemed unfeasible, for both orthopaedic surgeons and physiotherapists involved in patient care. Additionally, loss to follow-up and missing data are potential risks in prospective studies that the study team intends to minimize and that will be accounted for in data analysis. While this study focuses on patients with a TAS ≥ 7 with the desire to go back to the same level of sport, this subgroup was selected due to the sporting demands placed on the ACL graft. We do not claim that findings will universally apply to all ACL patients, and we understand that this may limit generalizability to lower-activity populations. However, we aim to generate insights that may contribute to evidence-based clinical decision-making and graft selection in athletic populations. Another limitation is that neither preoperative nor postoperative rehabilitation will be formally monitored or recorded. However, both follow standardized protocols routinely applied in our clinical practice.

Finally, the decision not to stratify randomization by sex could introduce imbalance between groups. However, the use of simple randomization is considered methodologically sound, with minimal risk of meaningful imbalance when sample sizes are 200 participants or larger [34, 60]. We plan to adjust for important covariates in the statistical analysis if imbalances are observed at baseline [60].

### Trial status

The inclusion of the first participant was on February 10, 2020, and the first intervention took place on May 13, 2020. Our intention was to prospectively register this trial and to fulfill the ICMJE guidelines, thus the trial was registered at ClinicalTrials.gov on February 6, 2020, prior to the inclusion of the first participant. However, a technical delay led to the protocol not being released for review until February 26, 2020, and not making it publicly available until March 4, 2020. Although the trial started at the beginning of the COVID-19 pandemic, it has continued and had a steady recruitment during and after the COVID-19 public health emergency. We have until now recruited 170 participants and hope to finalize inclusion at the end of 2025 or beginning of 2026. The estimated primary study completion date for 6 months analysis is therefore around the second half of 2026 or beginning of 2027.

## Data Availability

No datasets were generated or analysed during the current study.

## Abbreviations

ACL: Anterior cruciate ligament
ACL-R: Anterior cruciate ligament reconstruction
ACL-RSI: Anterior cruciate ligament Return to Sports Injury scale
ADL: Activity daily living
ATT: Anterior tibial translation
BPTB: Bone-patellar tendon-bone
DMC: Data Monitoring Committee
EQ-5D: European Quality of Life 5 dimension score
HT: Hamstrings tendon
ICMJE: International Committee of Medical Journal Editors
ICRS: International Cartilage Repair Society Classification
IKDC-SKF: International Knee Documentation Committee Subjective Knee Form
KOOS: Knee Osteoarthritis and Outcome Scores
LSI: Limb symmetry index
MRI: Magnetic resonance imaging
OA: Osteoarthritis
PROM: Patient reported outcome measure
RCT: Randomized clinical trial
OSTRC: Oslo Sports Trauma Research Center
ROM: Range of motion
QT: Quadriceps tendon
SPIRIT: Standard Protocol Items Recommendations for Interventional Trials 2013 Statement
QoL: Quality of Life
ST: Semitendinosus tendon
STS: Side to side
